# Antibiotic treatment duration for isolated methicillin-susceptible *Staphylococcus aureus* native tricuspid valve endocarditis: a standardized multidisciplinary approach

**DOI:** 10.1093/jacamr/dlaf171

**Published:** 2025-09-22

**Authors:** Sami El-Dalati, Bennett Collis, Evan Hall, Talal Alnabelsi, Chloe Cao, Meredith Johnson, John Gurley, Luke Strnad, Hassan Reda, Michael Sekela, Armaghan-E Rehman Mansoor, David Olafsson, William Harris, Bobbi Jo Stoner

**Affiliations:** Division of Infectious Diseases, Department of Internal Medicine, University of Kentucky Medical Center, Lexington, KY, USA; University of Kentucky College of Medicine, Lexington, KY, USA; University of Kentucky College of Medicine, Lexington, KY, USA; Division of Cardiology, Department of Internal Medicine, University of Kentucky Medical Center, Lexington, KY, USA; Department of Internal Medicine, HCA Healthcare/USF Morsani College of Medicine GME/HCA Florida Trinity, Tampa, FL, USA; Department of Surgery, University of Kentucky Medical Center, Lexington, KY, USA; Division of Cardiology, Department of Internal Medicine, University of Kentucky Medical Center, Lexington, KY, USA; Division of Infectious Diseases, Department of Internal Medicine, Oregon Health and Science University, Portland, OR, USA; Division of Cardiac Surgery, University of Kentucky Medical Center, Lexington, KY, USA; Division of Cardiac Surgery, University of Kentucky Medical Center, Lexington, KY, USA; Division of Infectious Diseases, Department of Internal Medicine, University of Kentucky Medical Center, Lexington, KY, USA; Division of Infectious Diseases, Department of Internal Medicine, University of Kentucky Medical Center, Lexington, KY, USA; University of Kentucky College of Pharmacy, Lexington, KY, USA; University of Kentucky College of Pharmacy, Lexington, KY, USA

## Abstract

**Background:**

MSSA tricuspid valve infective endocarditis (TVIE) is associated with significant morbidity and there is not consensus surrounding the optimal duration of antibiotic therapy. We report the outcomes of treating MSSA TVIE endocarditis using a multidisciplinary team and cardiovascular infectious diseases consult service.

**Methods:**

Patients were identified from the multidisciplinary endocarditis team registry in a single-centre retrospective study.

**Results:**

Between 7 September 2021 and 7 September 2024, 34 patients, including 27 who inject drugs, with isolated native MSSA TVIE were identified. Patients received a median of 28 days (IQR 11–41) of antibiotics and 50% transitioned to oral antibiotics for a median of 14 days. There was one relapsed infection (2.9%) and one death at 90 days (2.9%). There was no difference in relapsed infection or mortality at 90 days for patients transitioned to oral therapy compared with IV (*P* = 0.52).

**Conclusions:**

With a standardized approach to patient care, including a cardiovascular infectious diseases consult service and multidisciplinary team, patients with MSSA isolated TVIE without metastatic osteoarticular or spinal infections were successfully treated with antibiotic courses of ≤28 days with low rates of mortality and relapsed infection at 90 days.

## Introduction

Isolated native tricuspid valve infective endocarditis (TVIE) accounts for 5%–10% of all cases of infective endocarditis (IE) and is associated with lower mortality than left-sided IE.^1^ Up to 90% of patients with TVIE may have a history of injection drug use (IDU) and rates of IDU-related TVIE have increased over the last 15 years, correlating with the ongoing opioid epidemic.^2,3^  *Staphylococcus aureus* is the causative pathogen in 60%–90% of all TVIE and the proportion of cases caused by MSSA varies regionally.^4^ Treatment of MSSA TVIE involves antibiotic therapy and, in selected cases, procedural intervention, ideally guided by a multidisciplinary endocarditis team (MDET).^5,6^ Despite MSSA representing one of the most common causes of TVIE, the optimal duration and route of antibiotic administration has not been definitively established. While the American Heart Association (AHA) recommends 6 week courses of treatment for all cases of MRSA IE, regardless of the valve involved, there is less consistency regarding treatment duration for MSSA TVIE.^5,6^ The 2015 AHA guideline recommends 6 weeks of IV antibiotic therapy for most cases of MSSA TVIE but makes a comment that for patients with uncomplicated MSSA TVIE (individuals with no evidence of renal failure, extrapulmonary metastatic infections, meningitis, or aortic or mitral valve involvement) providers can consider treating for as short a duration as 2 weeks. Notably, this recommendation is also 2 weeks shorter than the 4 weeks of treatment that are recommended by the Infectious Diseases Society of America for complicated *S. aureus* bacteraemia.^7^ In contrast, the 2022 AHA scientific statement on endocarditis in people who inject drugs (PWID) advocated against the use of abbreviated 2 week regimens.^8^ The European society of Cardiology (ESC) guideline recommends treatment duration of 4–6 weeks for all cases of MSSA IE, regardless of which valves are involved, and does not provide specific guidance about how to select between durations.^5^ The ESC guideline also advocates for transitions to oral antibiotics after 10 days of IV therapy in stable patients.^9^ However, the ESC recommendations for oral treatment of MSSA IE include four-times-daily dosing with 1 g of dicloxacillin, which poses challenges for patient compliance, or the use of rifampicin, which can have substantial drug–drug interactions, particularly for patients on buprenorphine or methadone.^10^

These inconsistent recommendations from professional societies can lead to a range of treatment plans in clinical practice. Some patients may be offered 2 weeks of treatment or partial oral therapy after 10 days of IV antibiotics and others may be treated with 6 weeks of IV therapy. This variability can be particularly impactful for PWID, many of whom are deemed not suitable to complete outpatient parenteral antimicrobial therapy (OPAT) and are required to remain in the hospital or a monitored setting to complete treatment.^11,12^ In addition to higher costs, longer duration of therapy or inpatient IV antibiotic administration may impact patient experience as the rate of self-directed discharge is four times higher in cases of IDU-related IE compared with non-IDU-related IE.^13^ Clearly more data about treatment route and duration for MSSA TVIE are needed to guide providers, reduce costs and improve patient experience and clinical outcomes.

In this single-centre, retrospective cohort study, we report the 3 year experience of treating MSSA isolated native TVIE using a cardiovascular infectious diseases consult service (CVIDCS) and MDET.

## Methods

### Team protocol

In September 2021, the University of Kentucky Healthcare created an MDET and a CVIDCS.^14^ The composition, structure and day-to-day activity of the CVIDCS and MDET have previously been described.^15–18^ The MDET comprises providers from infectious diseases, cardiac surgery, cardiology, addiction medicine, neurosurgery, neurology, physical medicine and rehabilitation, palliative care and ethics. The group developed an internal protocol for medical treatment and formally meets weekly to discuss all inpatients with IE and document its recommendations in the electronic medical record. Cases for the conference are identified primarily by the CVIDCS in collaboration with the cardiac surgery service. Frequent communication between MDET providers and clinical primary teams also occurs outside of scheduled meeting times. The CVIDCS is an interdisciplinary team housed in the division of infectious diseases and comprises an attending physician, advanced practice provider, nurse navigator, pharmacist and social worker. The CVIDCS sees all inpatients with IE and follows them throughout their hospitalization and transition to outpatient. The CVIDCS coordinates the weekly MDET meetings and works with other specialties to schedule follow-up. All patients admitted to University of Kentucky Healthcare with TVIE are discussed at the weekly MDET conference. All patients with active substance use disorder or a history of IDU are offered addiction medicine consultation. Decisions regarding antibiotic route and duration, percutaneous mechanical aspiration and valve surgery are made at the MDET weekly meeting. The authors’ multidisciplinary approach to procedural intervention for native TVIE has been previously outlined.^16,17^ Surgical intervention is generally not performed during the index hospitalization, and percutaneous mechanical aspiration is used as a salvage therapy in patients with persistently positive blood cultures despite maximal medical therapy. In 2021, the CVIDCS created, implemented and published a standardized protocol for IV antibiotic therapy for IE and for transitioning patients with IE to oral antibiotic therapy.^19^ For MSSA IE specifically, all patients are initially treated with IV cefazolin or nafcillin. For patients with anaphylactic allergies to these medications, allergy and immunology consultation is requested for desensitization given the improved clinical outcomes for MSSA bacteraemia when treated with anti-staphylococcal β-lactams compared with vancomycin.^20,21^ Patients whose blood cultures are persistently positive for ≥72 h are started on combination therapy with ertapenem for synergistic killing of MSSA.^22,23^ This regimen is preferred to other combination therapies due to the existing literature supporting this approach and based on the clinical experience of the clinicians on the CVIDCS. Dual therapy is continued until two consecutive blood cultures are negative for 48–72 h, at which time ertapenem is discontinued. In general, all patients with MSSA isolated native TVIE who meet the AHA criteria for uncomplicated disease are treated with 4 weeks of antibiotic therapy. Individuals who leave the hospital via patient-directed discharge are typically provided with two additional weeks of oral antibiotic therapy and a follow-up appointment in the infectious diseases clinic within that time frame.

### Patient identification

Beginning in September 2021, a registry was created in the hospital’s electronic medical record containing all patients presented at the weekly MDET conference. Institutional review board approval was obtained from the University of Kentucky to establish a database to retrospectively collect each patient’s demographic, comorbidities, diagnostics, treatments and outcomes data. Patient consent was not required. For patients who did not follow-up in clinic, data for mortality and re-infections were collected based on emergency department visits and admissions to either the authors’ institution or other hospitals in the region.

Patients were included in this study if they met modified Duke criteria for definite MSSA native TVIE.^24^ Patients with extra-cardiac prosthetic material and concurrent osteomyelitis were included. Patients were excluded if they had evidence of left-sided IE, any prosthetic valve, a cardiac implantable electronic device (CIED) or left ventricular assist device (LVAD). The primary outcomes included in-hospital and 90 day mortality, as well as rates of relapsed infection at 90 days. Secondary outcomes included 30 and 90 day readmission rates.

The reporting of this study conformed to the Strengthening the Reporting of Observational Studies in Epidemiology statement.^25^

### Definition of terms

Active injection drug use was defined as patient-reported injection substance use within 30 days of the index hospitalization. Persistent bacteraemia was defined as blood cultures that were positive for ≥72 h from the initial positive culture.^23^ Blood culture clearance was defined by the presence of two consecutive negative blood cultures obtained on separate calendar days. The start date for treatment duration was defined from the date of blood culture clearance. Relapsed infection was defined by the presence of a new positive blood cultures with MSSA after previously documenting negative cultures.

Duration of oral antibiotic treatment was confirmed with patients at follow-up. For those who did not follow-up, duration was assumed, based on the number of days they received in the hospital plus the length of the oral antibiotic prescription, only if pick-up of the prescription was confirmed with the hospital’s pharmacy.

Consolidation antibiotic therapy was defined as oral antibiotic therapy provided for patients with MSSA vertebral osteomyelitis with the aim of preventing relapsed infection.

Acute renal failure was defined as patients who were initiated on renal replacement therapy during the index hospitalization.

## Ethics

This study was approved by the University of Kentucky Institutional Review Board (IRB #71514). Patient consent was not required by the IRB.

## Results

### Study population

**Figure 1.**
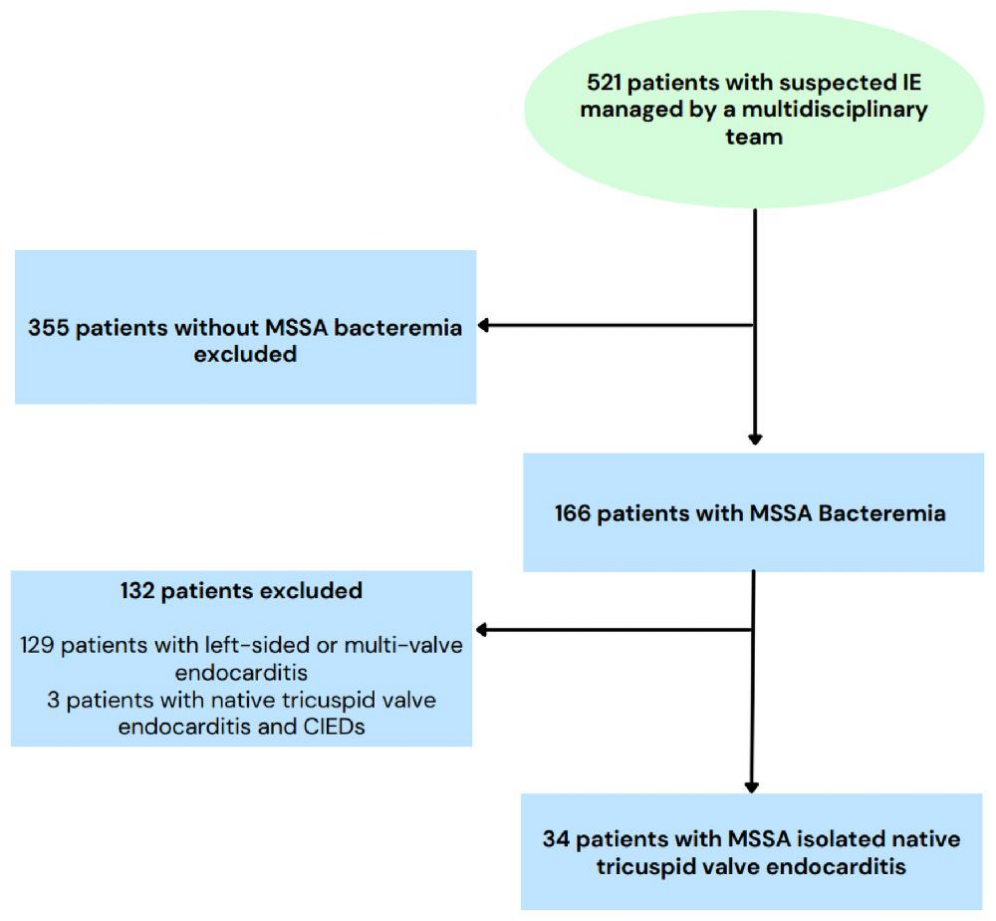
Study enrollment flowchart.

Between 7 September 2021 and 7 September 2024, 34 patients with definite MSSA isolated native TVIE were identified (Figure 1). Median patient age was 35.5 years (IQR: 29.3–40.8), 52.9% (*n* = 18) were female and 88.2% (*n* = 30) were Caucasian (Table 1). Seventy-nine percent (*n* = 27) had IDU and 8 (23.5%) had previous IE. Six patients (17.6%) were transferred from other institutions. Two-thirds of patients had persistent bacteraemia for ≥72 h with a median Pittsburgh bacteraemia score of 1 (IQR: 0–3). Patients were generally critically ill, and over half (55.9%; *n* = 19) were admitted to the ICU for a median stay of 9 days (IQR: 4.5–13.5). There were also substantial rates of vasopressor requirements (44.1%) and mechanical ventilation (32.4%). Seven patients (20.6%) had concurrent vertebral osteomyelitis, five (14.78%) had sacroiliitis and three (8.8%) had spinal epidural abscesses. There were no other documented sites of deep-seated metastatic infection and one patient had a concurrent prosthetic joint that was deemed not to be infected.

**Table 1.** Demographic information of patients with MSSA native TVIE managed by a multidisciplinary team

Variable	*n* = 34
Age (years), median (IQR)	35.5 (29.3–40.8)
Male, % (*n*)	47.1 (16)
Female, % (*n*)	52.9 (18)
Caucasian, % (*n*)	88.2 (30)
African American, % (*n*)	5.9 (2)
Previous infective endocarditis, % (*n*)	23.5 (8)
Active IDU, % (*n*)	79.4 (27)
Outside hospital transfer, % (*n*)	17.6 (6)
Chronic dialysis, % (*n*)	0 (0)
Diabetes, % (*n*)	8.8 (3)
Dental disease, % (*n*)	35.3 (12)
Hepatitis C viraemia, % (*n*)	52.9 (18)
Pregnancy, % (*n*)	0 (0)
Persistent bacteraemia, % (*n*)	67.6 (23)
Pittsburgh bacteraemia score, median (IQR)	1 (0–3)
ICU stay, % (*n*)	55.9 (19)
ICU stay (days), median (IQR)	9 (4.5–13.5)
Acute renal replacement, % (*n*)	11.8 (4)
Vasopressor requirement, % (*n*)	44.1 (15)
Mechanical ventilation, % (*n*)	32.4 (11)
Acute heart failure, % (*n*)	26.5 (9)
Septic pulmonary emboli, % (*n*)	79.4 (27)
Sacroiliitis, % (*n*)	14.7 (5)
Vertebral osteomyelitis, % (*n*)	20.6 (7)
Spinal epidural abscess, % (*n*)	8.8 (3)
Percutaneous mechanical aspiration, % (*n*)	2.9 (1)
Tricuspid valve replacement, % (*n*)	0 (0)

### Patient management

All patients were seen by the CVIDCS, discussed by the MDET, and initially treated with IV antibiotics for a median duration of 25 days (IQR: 11–41; Table 2). Cefazolin was the most commonly used IV antibiotic, administered in 27 patients (79.4%). Eight patients (23.5%) received nafcillin and one patient was treated with daptomycin. Two patients who were initially treated with nafcillin were changed to cefazolin or daptomycin after blood culture clearance due to adverse drug reactions. Over half (52.9%; *n* = 18) of patients received combination therapy with ertapenem for a median duration of 4 days (IQR: 3–5) before blood culture clearance. Only one patient received combination therapy with gentamicin. One patient underwent percutaneous mechanical aspiration and no patients received valve surgery during the index hospitalization.

**Table 2.** Antibiotic management for patients with MSSA native TVIE managed by a multidisciplinary team

Variable	*n* = 34
IV antibiotics, % (*n*)	100 (34)
Duration of IV antibiotics (days), median (IQR)	24 (11–41)
IV cefazolin, % (*n*)	79.4 (27)
IV nafcillin, % (*n*)	23.5 (8)
IV daptomycin, % (*n*)	2.9 (1)
Combination antibiotic therapy	55.8 (19)
Duration of combination antibiotic therapy (days), median (IQR)	4 (3–5)
Ertapenem coadministration, % (*n*)	52.9 (18)
Gentamicin coadministration, % (*n*)	2.9 (1)
Oral antibiotics for course completion, % (*n*)	50.0 (17)
Duration of oral therapy, % (*n*)	14 (12.5–14)
Linezolid, % (*n*)	17.6 (6)
Cefalexin, % (*n*)	5.9 (2)
Linezolid with cefadroxil, % (*n*)	23.5 (8)
Linezolid with rifampin, % (*n*)	2.9 (1)
Total duration of antibiotic therapy (IV and oral) (days), median (IQR)	28 (11–41)

Seventeen patients (50%) were transitioned to oral antibiotics for a median of 14 days (IQR: 12.5–14) to complete treatment. Twelve of these individuals were switched due to patient-directed discharge. Fifteen of these patients (88.2%) received linezolid, including eight who received coadministered cefadroxil and one who received coadministered rifampin. Six patients received linezolid monotherapy and two were treated with cefalexin alone. The median total duration of antibiotic therapy (IV plus oral) for treatment of the MSSA isolated native TVIE was 28 days (IQR: 11–41). Six patients (17.6%) received consolidation therapy for a median of 92.5 days (IQR: 92–93) for co-occurring osteomyelitis.

### Patient outcomes

Median length of stay was 21.5 days (IQR: 11–39.3) and there was one in-hospital death after 12 days of antibiotic therapy, giving an in-hospital mortality of 2.9% (Table 3). This patient was diagnosed with endocarditis at another institution before a patient-directed discharge and then later presented to our institution in multi-organ failure. There were no additional deaths at 90 days. There was one relapsed infection at 30 days in a patient who left self-directed after receiving 2 days of IV antibiotics, was not able to take the prescribed oral antibiotics and returned to the hospital with recurrent MSSA bacteraemia. There were no additional relapsed infections between 30 and 90 days post discharge. Twelve patients (35.3%) left the hospital via patient-directed discharge. There were 12 (35.3%) readmissions at 90 days and 11 of these (92.9%) occurred within 30 days of discharge. Five (41.6%) of the 90 day readmissions occurred in patients who left self-directed. Approximately 60% (*n* = 20) of patients were followed up in infectious diseases clinic. Thirteen patients (38.2%) had no follow-up at the authors’ institution.

**Table 3.** Clinical outcomes for patients with MSSA native TVIE managed by a multidisciplinary team

Variable	*n* = 34
Length of stay (days), median (IQR)	21.5 (11–39.3)
Patient-directed discharge, % (*n*)	35.3 (12)
Inpatient mortality, % (*n*)	2.9 (1)
90 Day mortality, % (*n*)	2.9 (1)
30 Day relapsed infections, % (*n*)	2.9 (1)
30 Day readmissions, % (*n*)	32.4 (11)
90 Day relapsed infections, % (*n*)	2.9 (1)
90 Day readmissions, % (*n*)	35.3 (12)

### IV versus oral antibiotic treatment

Ten individuals (29.4%) received 6 weeks of exclusively IV antibiotic therapy (Table 4). Nine of these patients had evidence of vertebral osteomyelitis, sacroiliitis or spinal epidural abscesses. The one patient who did not had underlying cirrhosis. Patients who received 6 weeks of IV antibiotics were more likely to be male compared with patients who received <6 weeks of IV treatment (80% versus 33.3%; *P* = 0.01). There were more patient-directed discharges among patients who received <6 weeks of IV antibiotics (45.8% versus 10%; *P* = 0.05) and median length of stay was significantly longer among patients who received 6 weeks of IV treatment (44 versus 12.5 days; *P* = 0.001). Otherwise the two groups were similar with respect to demographics and illness acuity. There were no significant differences in in-hospital and 90 day mortality, 90 day relapsed infection or 90 day all-cause readmission between patients who received <6 weeks of IV antibiotics or 6 weeks of IV treatment.

**Table 4.** Comparison of patients with MSSA native TVIE treated with 6 weeks of IV antibiotics and patients treated with <6 weeks of IV antibiotics

Variable	6 Weeks of IV antibiotics *n* = 10	Less than <6 weeks of IV antibiotics *n* = 24	*P* value
Duration of IV antibiotics (days), median (IQR)	41.5 (41–42)	13 (4–24.5)	*P* < 0.0001
Synergism with ertapenem, % (*n*)	60.0 (6)	50.0 (12)	0.60
Oral antibiotic for course completion, % (*n*)	10.0 (1)	66.7 (16)	0.0030
Persistent bacteraemia, % (*n*)	70.0 (7)	66.7 (16)	0.85
Pittsburgh bacteraemia score, median (IQR)	1 (0–2.8)	1.5 (0–3)	0.85
Prior infective endocarditis, % (*n*)	10.0 (1)	29.2 (7)	0.23
Age (years), median (IQR)	40 (31.3–45)	35 (29.8–40)	0.28
Male, % (*n*)	80.0 (8)	33.3 (8)	0.01
Female, % (*n*)	20.0 (2)	66.7 (16)	0.01
Caucasian, % (*n*)	90.0 (9)	87.5 (21)	0.84
African American, % (*n*)	10.0 (1)	4.2 (1)	0.52
IDU, % (*n*)	70.0 (7)	83.3 (20)	0.39
Hepatitis C viraemia, % (*n*)	50.0 (5)	54.2 (13)	0.83
Dental disease, % (*n*)	20.0 (2)	41.7 (10)	0.23
Diabetes, % (*n*)	20.0 (2)	4.2 (1)	0.15
Acute heart failure, % (*n*)	30.0 (3)	25.0 (6)	0.77
Septic pulmonary emboli, % (*n*)	70.0 (7)	83.3 (20)	0.39
Epidural abscess, % (*n*)	10.0 (1)	8.3 (2)	0.88
Vertebral osteomyelitis, % (*n*)	40.0 (4)	12.5 (3)	0.08
ICU stay, % (*n*)	50.0 (5)	58.3 (14)	0.66
ICU stay (days), median (IQR)	10 (7–14)	8.5 (4.3–12.8)	0.51
Acute renal replacement therapy, % (*n*)	20.0 (2)	8.3 (2)	0.34
Vasopressor, % (*n*)	40.0 (4)	45.8 (11)	0.76
Mechanical ventilation, % (*n*)	40.0 (4)	29.2 (7)	0.55
Patient-directed discharge, % (*n*)	10.0 (1)	45.8 (11)	0.05
Length of stay (days), median (IQR)	44 (31–45.8)	12.5 (9.8–26.3)	0.001
In-hospital mortality, % (*n*)	0 (0)	4.2 (1)	0.52
90 Day mortality, % (*n*)	0 (0)	4.2 (1)	0.52
30 Day relapsed infection, % (*n*)	0 (0)	4.2 (1)	0.54
30 Day all-cause readmission, % (*n*)	20.0 (2)	37.5 (9)	0.33
90 Day relapsed infection, % (*n*)	0 (0)	4.2 (1)	
90 Day all-cause readmission, % (*n*)	30.0 (3)	37.5 (9)	0.68

## Discussion

This single-centre retrospective cohort study reports on 34 patients with MSSA isolated native TVIE managed by a CVIDCS and MDET. While other, older studies, have reported on outcomes for MSSA TVIE, our study is unique in that it incorporates the use of a multidisciplinary team and reports on a standardized approach to choosing treatment route and duration. There are several notable findings from this study. The median total duration of antibiotic therapy was 28 days, which is 2 weeks shorter than the 6 week duration of therapy often recommended for MSSA isolated native TVIE.^5,6^ In-hospital and 90 day mortality rates were very low despite high rates of ICU admission, mechanical ventilation and vasopressor support. Half of patients were transitioned to oral antibiotics, allowing them to complete therapy outside of the hospital without evidence of inferior clinical outcomes. This is a particularly important finding as the previously published literature on partial oral antibiotic treatment of endocarditis has primarily focused on left-sided disease.^9,26^ There were very low rates of percutaneous mechanical aspiration and no valve surgeries, consistent with the authors’ previously published approach to management of native TVIE.^16,17^

The 28 day median duration of antibiotic therapy is notable since half of patients received <4 weeks of treatment and one-quarter received ≤11 days of antibiotics. This was driven primarily by a high rate of patient-directed discharge (35.3%) and all of these occurred in patients with a history of substance use. This occurred even with a dedicated addiction medicine consult service at the study site. While addiction medicine teams have been shown to be effective at initiating medications for opioid use disorder and reducing mortality, treatment of symptoms of acute withdrawal is often managed at our hospital by the primary admitting service.^27,28^ Published literature suggests that undertreatment of withdrawal and stigma from hospital staff may contribute to patient-directed discharge.^29^ Other factors affecting discharge may include competing life priorities such as maintaining shelter or caring for children.^29^ There were equal numbers of patient-directed discharges amongst male and female patients (six each). Notably, more male patients were treated with 6 weeks of IV antibiotics but it is difficult to draw definitive conclusions from this finding given the small sample size. Although it is also not possible to conclude in this study exactly what contributed to the high rates of patient-directed discharge, it is clear there is substantial room to improve in this area.

Six patients left the hospital after receiving ≤4 days of IV antibiotics. Despite this, 90 day mortality and relapsed infection rates remained low. This was likely driven by the practice of providing prescriptions for 14 days of oral antibiotics for self-directed discharges. Previous data have demonstrated a mortality benefit to providing oral antibiotics at the time of patient-directed discharge for patients with *S. aureus* bacteremia.^30^ It appears that a subset of patients can be successfully treated with approximately 2 week courses of antibiotics, as previously demonstrated and recommended by the AHA.^6,31^ The only relapsed infection in the cohort occurred in a patient who received only 2 days of IV antibiotic therapy, left self-directed and did not take the prescribed oral antibiotics.

Notably, there were 11 patients who had vertebral complications of their endocarditis defined by the presence of sacroiliitis, epidural abscess and/or vertebral osteomyelitis. These patients were treated for longer, receiving a median duration of 41 days of IV antibiotic therapy compared with 28 days for the entire cohort. Four of these 11 patients (36.4%) were also transitioned to consolidation oral antibiotics for a median duration of 92.5 days.

One particular strength of this study is the standardized management approach to patients with isolated native TVIE. All patients were seen by infectious diseases providers who primarily specialize in treatment of endovascular infections. The approach to antibiotic therapy was driven by a standardized, written protocol and all patients were initially treated with IV anti-staphylococcal β-lactams, and combination antibiotic therapy with ertapenem was utilized in over half of patients. Percutaneous mechanical aspiration was pursued in cases of persistent bacteraemia refractory to medical therapy and was required in only one patient. Transitions to oral antibiotic therapy were also protocol-driven using an internal, evidence-based guideline, and primary teams were provided with recommendations for oral antibiotics in the event of patient-directed discharge. Our institutional oral protocol, which is adapted from Iversen *et al.*, recommends treating patients with two different oral agents with different mechanisms of action.^9,19^ Our clinical experience attempting to use dicloxacillin found that most patients could not tolerate taking 1 g of dicloxacillin four times per day due to gastrointestinal side effects and the difficulty adhering to such frequent dosing. Consequently, we amended our approach and substituted cefadroxil for dicloxacillin. In general, if patients discharged with <7 days of antibiotic therapy remaining, they were treated with oral linezolid monotherapy, and if discharging with >7 days of antibiotic therapy, they were treated with both oral linezolid and cefadroxil. Follow-up appointments were also made prior to discharge and patients were provided with the contact information for the infectious diseases clinic and CVIDCS nurse navigator. However, as noted above, even with this standardized management approach there were substantial rates of patient-directed discharge, readmission and 38% of patients did not attend any follow-up appointments at the authors’ institution.

### Limitations

Our study is limited by its retrospective, single-centre design and the relatively small number of patients. Additionally, patients were only included if they were discussed by the MDET and it’s possible there were other patients with MSSA TVIE admitted to our institution that were excluded. Access to outside hospital records was limited and patients presented to our institution from a wide catchment area. Therefore, it’s possible that other post-discharge complications occurred and were not captured by study investigators. Patients with prosthetic valves, CIEDs and LVADs were excluded and our results cannot be extended to these populations.

### Conclusions

MSSA isolated native TVIE is a potentially life-threatening complication of IDU that is associated with significant morbidity and costs. Consensus guidelines do not provide clear recommendations on the ideal antibiotic treatment duration and as a result there can be variability in provider practice. Utilizing a standardized approach to patient care, including a CVIDCS and MDET, patients with MSSA isolated TVIE without metastatic osteoarticular or spinal infections were successfully treated with antibiotic courses of ≤28 days with low rates of mortality and relapsed infection at 90 days and notable savings in IV access and hospital days compared with treatment courses of 6 weeks. More research is needed to identify ways to mitigate patient-directed discharges and reduce rates of readmission.

## Data Availability

The data that support the findings of this study are available on request from the corresponding author. The data are not publicly available due to privacy or ethical restrictions.
